# Co-administration of Spirulina and L-carnitine preserves ovarian reserve in a rat model of premature ovarian insufficiency via SIRT1 regulation of oxidative stress, inflammation, and apoptosis

**DOI:** 10.1038/s41598-025-11143-2

**Published:** 2025-09-15

**Authors:** Eman M. Embaby, Gehad E. Elshopakey, Aya Megahed, Shaymaa Rezk, Ahmed Ateya, Mamdouh Eldesoqui, Eman H. Yousef, Mona M. Elghareeb

**Affiliations:** 1 Department of Physiology, Faculty of Veterinary Medicine, Mansoura University, Mansoura, 35516 Egypt; 2 Department of Clinical Pathology, Faculty of Veterinary Medicine, Mansoura University, Mansoura, 35516 Egypt; 3 Department of Veterinary Diseases, Faculty of Veterinary Medicine, Delta University for Science and Technology, Gamasa, 35712 Egypt; 4 Department of Cytology and Histology, Faculty of Veterinary Medicine, Mansoura University, Mansoura, 35516 Egypt; 5 Department of Development of Animal Wealth, Faculty of Veterinary Medicine, Mansoura University, Mansoura, 35516 Egypt; 6 Department of Basic Medical Sciences, College of Medicine, AlMaarefa University, P.O.Box 71666, Riyadh, 11597 Saudi Arabia; 7 Department of Anatomy and Embryology, Faculty of Medicine, Mansoura University, Mansoura, 35516 Egypt; 8 Department of Pharmacology and Biochemistry, Faculty of Pharmacy, Horus University-Egypt, Damietta, Egypt

**Keywords:** Spirulina, L-carnitine, Premature ovarian insufficiency, SIRT1, Oxidative stress/Inflammation, Apoptosis, Reproductive disorders, Histocytochemistry, Hormones

## Abstract

This study aimed to assess the possible consequences of spirulina (SP) and/or L-carnitine (L-car) on the prevention of primordial follicular reserve depletion and the preservation of ovarian follicular structure and function in a rat model of premature ovarian insufficiency (POI). Forty healthy adult female Sprague-Dawley albino rats were randomly assigned into five equal-sized groups (*n* = 8): normal control, DOX group (2.5 mg/kg i.p.), SP group (500 mg/kg intragastric), L-car group (250 mg/kg i.p.), and SP + L-car group in the same previous doses. In comparison to the DOX group, administration of either SP or L-car significantly increased serum E2 and AMH levels along with a significant decrease in the FSH and LH levels (*p <* 0.05). The oxidative stress was significantly reduced. Ovarian expressions of NF-κB, iNOS, FOXO1, P53, and caspase-3 decreased significantly, while SIRT1, STAR, CYP17A1, HSD17B3, Nrf2, and mtDNA increased significantly. Histology revealed histoarchitecture improvement as the mean % of atretic follicles and degenerated corpora lutea was significantly reduced (*p <* 0.05). The combined treatments synergistically improved the parameters studied more than either treatment alone. The molecular docking results revealed the ability of both n-hexadecanoic acid and L-carnitine to activate SIRT1 and subsequent antioxidant, anti-inflammatory, and anti-apoptotic pathways. This ovoprotective effect is suggested to be mediated through activation of different SIRT1-mediated protective signaling pathways that remodel ovarian redox status, inflammation, and apoptosis, which may strengthen the potential role of SP and L-car as a chemotherapy adjuvant, reducing the negative health effects of early menopause after cancer therapy.

## Introduction

Female reproduction is determined by ovarian folliculogenesis, a dynamic and tightly controlled process that includes primordial follicle assembling, follicle development, ovulation, and formation of the corpus luteum^1^. Severe ovarian pathological diseases, such as polycystic ovary syndrome and premature ovarian insufficiency (POI), are caused by anomalies in folliculogenesis^2,3^. Follicle atresia is the term for degradation that occurs in most follicles throughout growth and maturation. Atypical follicular atresia and primordial follicle stimulation may encourage follicular depletion, leading to early menopause and ovarian insufficiency^4^. The hypothalamic-pituitary-ovarian (HPO) axis is a sophisticated neurohormonal mechanism that controls the oocyte release sporadically during ovulation^5^. On the other hand, POI is defined by disruption of the HPO axis, which can lead to amenorrhea or oligomenorrhea, low levels of estradiol, and increased gonadotropin levels^6,7^.

Premature ovarian failure in female cancer survivors after chemotherapy has aroused widespread public concern. Unquestionably, doxorubicin (DOX) and other anticancer drugs are efficacious therapeutic interventions. Nonetheless, it is impossible to overlook their negative impact on the lives of female cancer survivors^8^. Reproductive system damage, which is often linked to POI, is one of the most disastrous consequences of chemotherapy^9–11^. The pathophysiology of DOX-induced POI is complex, but numerous factors have been proposed to be linked to the onset and development of ovarian dysfunction, including free radical-mediated lipid peroxidation, oxidative stress, mitochondrial malfunction, increased proinflammatory release of cytokines, and granulosa cell apoptosis^12–14^. Antioxidant and anti-inflammatory agents play a crucial role in protecting ovarian tissue from chemotherapy-induced damage by reducing oxidative stress, inflammation, and apoptosis^15,16^.

Recent studies have reported that silent information regulator 1 (SIRT1), a class III protein deacetylase, is crucial for the control of cellular survival^17^. By modulating many pathways (such as p53, FOXO, Nrf2, NF-κB), among other things, it can participate in the control of oxidative stress, inflammatory reactions, apoptosis, and the breakdown of energy. This makes it a significant factor in toxicological harm^18–20^. Researchers discovered that the POI model revealed lower ovarian SIRT1 expression, indicating a significant relationship between SIRT1 and ovarian reserve and its possible application as a measure of ovarian aging^21^. Consequently, we proposed practical uses of antioxidant therapy, targeting positive SIRT1 regulation, which will protect ovarian functions during DOX treatment and prevent subsequent induced POI.

Lately, the application of medicinal plants that contain antioxidants has attracted worldwide interest^22^. Spirulina (SP), blue-green algae, has been demonstrated as an antioxidant and anti-apoptotic in many in vitro and in vivo studies^23^. Arthrospira platensis, the most common genus of SP, is useful because their bioactive ingredients affect several biological signaling pathways; consuming it through food can help prevent or manage high blood sugar, high cholesterol, heart disease, diabetes, other metabolic disorders, certain inflammatory conditions, allergies, cancer, environmental pollutants, drug-induced toxicities, and infections caused by viruses^24–26^. Also, its protective effect on the rat ovary against DOX-induced POI has been published^27^. Several studies reported that SP displayed protective effects in rats’ liver and kidney against toxicities through upregulation of the SIRT1 expression^28,29^. So, SP has the potential to work in combination with medicinal medications to improve treatment outcomes^30^.

On the other hand, L-carnitine (L-car) is an essential quaternary amine produced naturally from methionine and lysine. It is mostly acquired by eating meat and other animal products^31^. L-car supplementation has been proven in clinical investigations to improve ovarian function by carrying long-chain fatty acids from the cytoplasmic area into the mitochondria, so they undergo oxidation to generate energy. In mammalian tissues, they also serve as scavengers of oxygen free radicals, effectively preventing oxidative stress-induced mitochondrial damage and mitochondria-dependent apoptosis in a variety of cell types^32–34^. A study reported that L-car displayed anti-inflammatory, antioxidant, and anti-apoptotic properties via regulating SIRT1 expression in the renal tissue of rats^35^. Hence, SIRT1 regulation may be related to the regulatory role of L-car against POI.

Based on the previous layout, we postulated that SP and L-car might mutually enhance their antioxidant capacities, ultimately providing a more prolonged protective effect than either could supply on its own. To the best of our knowledge, no study has looked at the possibility of a synergistic protective effect between SP and L-car and the involvement of ovarian SIRT1 regulation in their ameliorating mechanisms against POI. Thus, in an experimental model of DOX-induced POI in adult female albino rats, this research seeks to assess the possible consequences of SP and/or L-car on the prevention of primordial follicular reserve depletion, preservation of ovarian follicular structure and function, and improvement of long-term post-chemotherapy fertility utilizing immunohistochemistry, histology, biochemistry, and molecular studies.

## Materials and methods

### Experimental chemicals and drugs

Since doxorubicin, spirulina, and L-carnitine are commercially accessible, they were acquired at a nearby pharmacy in Egypt. DOX was acquired as an injection-grade vial (50 mg/50 mL, Mylan, Italy), SP as tablets (the active ingredient is included in 500 mg each pill, Puritan’s Pride Company, Inc., USA), and L-car as injection-grade ampoules (1 mg/5 mL/5 ampoules, Mepaco, Cairo). After being pulverized, the SP pills were taken orally as a fresh solution in 0.9% saline. Based on previously published research by Samare-Najaf, et al.^12^, Afkhami-Ardakani, et al.^36, 37^, respectively, the dose and delivery method of DOX, SP, and L-car were determined.

### Analysis of Spirulina (Arthrospira platensis) by gas Chromatography-Mass spectrometry (GC-MS)

**According to El-Kareem** et al.^38^making use of a TG-5MS capillary column (30 m x 0.25 mm x 0.25 μm film thickness) and a Trace GC-TSQ mass spectrometer (Thermo Scientific, Austin, TX, USA), the chemical composition of SP samples was determined. The temperature of the column oven was first maintained at 50 °C, then raised by 5 °C per minute to 250 °C and held for two min. Finally, it was raised to 300 °C by 30 °C per min and kept for two min. Helium was utilized as a carrier gas at a steady flow rate of 1 mL per min, while the temperature of the MS transfer line and injector was kept at 260 and 270 °C, respectively. Following a 4 min solvent delay, diluted specimens of 1 µl were mechanically injected using the Autosampler AS1300 coupled with GC in the split mode. EI mass spectra were acquired in full scan mode spanning the m/z 50–650 range at ionization voltages of 70 eV. The ion source’s temperature was set at 200 °C. The components were identified by matching their mass spectra to those of the WILEY 09 and NIST 14 mass spectral databases.

### In Silico molecular Docking (MD)

The binding affinity of n-hexadecanoic acid (the largest fatty acid contained in SP) and L-carnitine towards SIRT1 was modeled using Autodock Vina 1.1.2 ^39^. First, the Protein Data Bank (https://www.rcsb.org/pdb/home/home.do) was used to download the SIRT1 crystal structure (**PDB code: 4ZZJ**). Then, every water molecule and complex associated with this protein was removed. The addition of hydrogen changed the tautomeric states and ionization of amino acids. The three-dimensional structures of n-hexadecenoic acid and L-car were obtained from the ZINC database and chemically optimized by adding hydrogens, changing the bond ordering, and adding charges. Additionally, energy minimization was applied to the construction. To make the protein and ligand structures compatible with the AutoDock application, the PDBQT file format was used to convert them. AutoDock Vina was used to perform molecular docking at the 4TQ ligand attachment site. According to the X, Y, and Z dimensions in the grid map that was employed, the docking results were characterized as -0.423893, 44.524196, and − 0.038714 for the co-crystallized ligand, respectively. Finally, Chimaera 1.15 and Discovery Studio Visualizer version 21.1.0.20298 were used to analyze and visualize the docking postures and interactions between them inside the binding pocket.

### Sample size calculation

The sample size was determined using G*Power 3.1.9.4 software in accordance with the methodologies outlined by Faul et al.^40^referencing the prior work by Rad et al.^41^. The power study found that a sample size of *N* = 8 in each group provides more than 95% power (α = 0.05), ensuring methodological and statistical consistency. The comparatively higher sample size addresses biological variability, possible attrition and death rates (e.g., Doxorubicin toxicity), and type I error inflation in pairwise comparisons (Tukey’s post-hoc test).

### Animal care and handling

Forty healthy adult female Sprague-Dawley albino rats (180–220 g, 2 months old). Rats were obtained from the Laboratory Animal House at Mansoura University’s Faculty of Veterinary Medicine. Vaginal smear was daily obtained as described previously^42^and only those showing at least two consecutive normal vaginal estrus cycles (4–5 days) were used in the experiments. At Mansoura University’s Physiology Department, Faculty of Veterinary Medicine, the rats were kept in separate plastic cages, at the rate of six per cage, with wood shavings as bedding. Rats were acclimated for two weeks in a controlled setting with 12/12-hour light/dark cycles and a temperature of 25 ± 2˚C and humidity. Additionally, every experiment was carried out at the same time of day, from 8 a.m. to 2 p.m., to get around changes brought on by daily cycles. The rats were fed a balanced meal and given unrestricted access to water.

### Ethical approval of animal use

All experiment procedures were performed in accordance with the relevant guidelines and regulations of the animal welfare committee at the Faculty of Veterinary Medicine, Mansoura University, Egypt, and approved with registration code number: MU-ACUC (VM.R.24.09.178).

### Experimental design

**Fig. 1:**
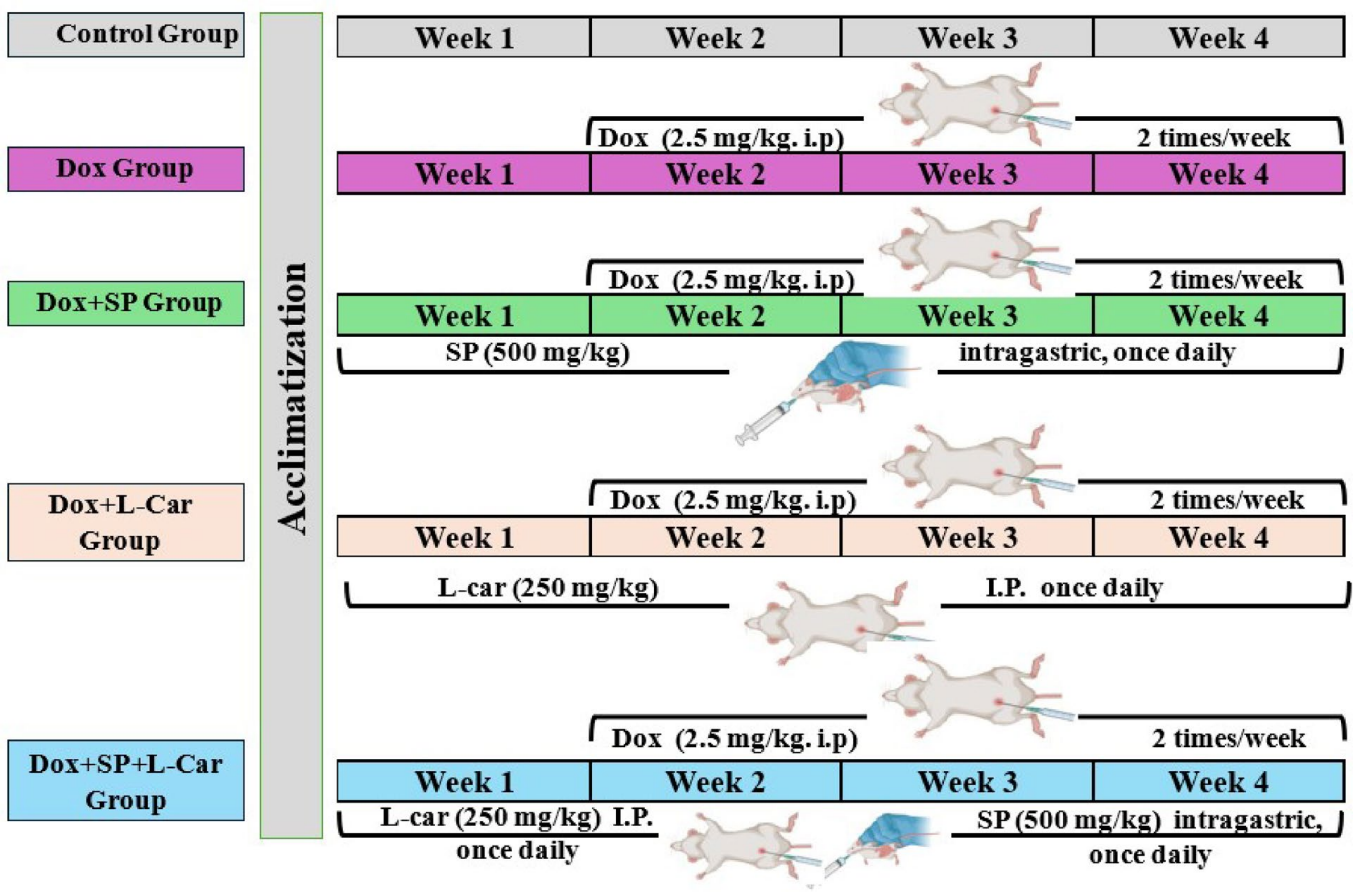
Experimental protocol (experimental groups, treatments, and dose of each treatment).

Five equal groups of eight cycled female rats each were randomly assigned to the following groups **(**Fig. 1**)**:

#### Control group

Rats received intraperitoneal (i.p.) injections of a 0.9% saline solution (2.5 ml/kg) for 28 consecutive days.

#### DOX group

To create the POI rat model, rats were given doxorubicin (DOX, 2.5 mg/kg; i.p.) on Mondays and Fridays for a total of three weeks in a row. This was done in six equal doses of 2.5 mg/kg each, for a total cumulative dosage of 15 mg/kg. Induction of POI was carried out in lesser doses and at certain intervals because the application of large quantities of DOX at once resulted in severe toxicity and fatal consequences^43^.

#### DOX + SP group

Before starting chemotherapy, rats were given spirulina (SP, 500 mg/kg; intragastric) once daily for seven days in a row. This treatment was maintained for 21 days in a row, during which time DOX was used to induce POI.

***DOX + L-car group*** Before starting chemotherapy, rats were given L-carnitine (L-car, 250 mg/kg; i.p.) once daily for seven days in a row. This treatment was maintained for 21 days in a row, during which time DOX was used to induce POI.

#### DOX + (SP + L-car) group

Prior to starting chemotherapy, the rats were given SP (500 mg/kg; intragastric) and L-car (250 mg/kg; i.p.) once daily for seven days in a row. This treatment was maintained for 21 days in a row, during which time DOX was used to induce POI.

### Sample collection

On the 28th day of the experiment, the animals were maintained in a starvation condition overnight. All rats were sacrificed the next day using sodium pentobarbital (Somnopentyl, 50 mg/kg, i.p.; Kyoritu Pharmaceutical Co., Ltd., Tokyo, Japan). Intracardiac blood samples (5 mL) were then promptly taken in sterile centrifuge tubes and left to coagulate at room temperature. Using a cooling centrifuge, samples of serum were centrifuged at 1800×g for 15 min at 4 °C for laboratory purposes. They were then stored at -20 °C until analysis. After a laparotomy, the ovaries were gently removed (a procedure known as a bilateral oophorectomy). While the left ovaries were flash-frozen and stored at -80 °C until they were utilized for biochemical and molecular research, the right ovaries were fixed in 10% buffered formalin for histopathological and immunohistochemical analyses.

### Biochemical analysis

#### Serum hormonal assays

Serum samples were subjected to hormonal tests for estradiol (E2), follicle-stimulating hormone (FSH), and luteinizing hormone (LH) utilizing chemiluminescent detection techniques using magnetic particle division (Beckman Coulter-Access 2 immunoassay system, Irving, TX, USA). Hormone levels in serum may be measured using a two-step, paramagnetic particle, chemiluminescent enzyme immunoassay that makes use of Access immunoassay technology. The unbound conjugate is removed in a second separation and washing phase. A luminometer is used to measure the quantity of light produced by the reaction once a chemiluminescent substrate called Lumi-Phos 530 has been added to the reaction vessel. The number of photons produced is directly correlated with the number of hormones present in the sample. The quantity of analyte in the sample is ascertained using a stored multi-point calibration curve. A dry fluorescent immunoassay based on an antigen-antibody reaction was used to quantify anti-mullerian hormone (AMH) in accordance with the kits purchased from Lansion Biotechnology Co., Ltd. (Nanjing, Jiangsu Province, China) after following the included pamphlet protocol.

#### Ovarian oxidant/ antioxidant status

Ovarian homogenate was subjected to measurements of lipid peroxidation and enzyme activity with commercial test kits from Biodiagnostic Co., Cairo, Egypt. According to Ohkawa, et al.^44^, the quantity of malondialdehyde (MDA) was measured in order to evaluate the levels of lipid peroxidation. In accordance with Green^45^ procedure, nitric oxide (NO) was also calculated calorimetrically using the Griess reagent. The ability of the antioxidants in a sample to block or diminish 2,2’-azinobis (3-ethylbenzothiazoline-6-sulfonic acid, or ABTS) and the blue-green ABTS radical (ABTS•+) that is formed is the broad basis for the total antioxidant capacity (TAC) test, which is measured at 505 nm. According to Sun et al.^46^, the ability of superoxide dismutase (SOD) to prevent the reduction of the nitroblue tetrazolium dye to diformazan, which is mediated by phenazine methylsulfate (PMS), was used to assess SOD activity. Moreover, catalase (CAT) activity was determined by measuring the decrease in H_2_O_2_ concentration after adding 10 µL of serum to the kit reagent^47^. Ellman’s reagent was used to demonstrate the glutathione (GSH) level, and the produced yellow chromogen was measured at 412 nm^48^.

## Gene analysis

### Reverse transcription and total RNA extraction of the target genes

Total RNA was obtained from ovary samples using the Trizol reagent in accordance with the manufacturer’s instructions (Direct-zol TM RNA MiniPrep, cat No. R2050). The amount and purity were tested using a Nanodrop (UV-Vis spectrophotometer Q5000/USA), and the integrity was evaluated using gel electrophoresis. Following the manufacturer’s instructions, the RNA samples were then reverse transcribed into cDNA (SensiFast™ cDNA synthesis kit, Bioline, cat. No. Bio-65053). The reaction mixture was carried out in a total volume of 20 µL consisting of total RNA up to 1 µg, 4 µL 5x Trans Amp buffer, 1 µL reverse transcriptase, and DNAase-free water up to 20 µL. After putting the finished reaction mixture in a heat cycler, the following procedure was run: primer annealing for 10 min at 25 °C, reverse transcription for 15 min at 42 °C, and inactivation for 5 min at 85 °C. At 4 °C, the samples were stored.

### Quantitative Real-time-PCR (qRT-PCR) analysis

The relative ovarian SIRT1, steroidogenic acute regulatory protein (STAR), cytochrome P450 17A1 (CYP17A1), hydroxysteroid (17β) dehydrogenase type 3 (HSD17B3), nuclear factor erythroid 2 related factor 2 (Nrf2), mitochondrial DNA (mtDNA), forkhead box protein O1 (FOXO1), nuclear factor kappa B (NF-κB), and inducible nitric oxide synthase (iNOS) mRNA abundance was determined by qRT-PCR using SYBR Green PCR Master Mix (2x SensiFast SYBR, Bioline, catalog No. Bio-98002). The primer sequence used for qRT-PCR analysis is shown in Table 1. As an internal control, the housekeeping gene β-actin was used. The reaction mixture, which had a total volume of 20 µL, contained 0.8 µL of each primer, 10 µL of 2x SensiFast SYBR, 3 µL of cDNA, and 5.4 µL of H_2_O (d.d. water). The following qRT-PCR conditions were used: 40 cycles of 94 °C for 15 s, annealing temperatures as indicated in Table (1) for 30 s, and extension temperature at 72 °C for 20 s were performed after 95 °C for 4 min. At the end of the amplification phase, a melting curve analysis was performed to confirm the specificity of the PCR product. To normalize target gene expression levels, the relative expression of the gene in each sample versus a control in comparison to the β-actin gene was calculated according to the 2-ΔΔCt method **(41)**.

**Table 1:** Primer sequence and melting temperature used in qRT-PCR.

Gene	GenBank accession number	Oligonucleotide sequence	Annealing temperature (^0^C)	Size (bp)
*SIRT1*	NM_001372090.1	f5,- TTCTGTTTCCTGTGGGATACCTG-3, r5, - TGCTCATGAATGCTGAGTTGC-3,	60	225
*STAR*	AF044081.1	f5,- GGGCATACTCAACAACCAG-3, r5,- ACCTCCAGTCGGAACACC-3,	58	111
*CYP17A1*	NM_012753.3	f5,- CTCTGGGCACTGCATCAC-3, r5,- CAAGTAACTCTGCGTGGGT-3,	58	114
*HSD17B3*	NM_054007.1	f5,- CTGGAAGCCGTGTGAAGGTT-3, r5,- CCTCTCCGCCTTGATTCCAT-3,	58	247
* Nrf2*	NM_001399173.1	f5, - CCACTGCTCCGACTAGCCAT-3, r5, - GCGGTGGCAATTCCAAGTCC -3,	60	115
*MtDNA*	OP149648.1	f5,- ATAAGACATCTCGATGGTAACG-3, r5, - CCACAGGACTTTGTGCTGACCT-3,	58	140
*NF-κB*	AF079314.2	f5,- TGGACGATCTGTTTCCCCTC − 3, r5,- CCCTCGCACTTGTAACGGAA-3,	59	118
*iNOS*	NM_012611.3	f5,- TGGGTGAAAGCGGTGTTCTT − 3, r5,- TAGCGCTTCCGACTTCCTTG − 3,	60	108
*FOXO1*	NM_001191846.3	f5,- CCGCGTCTCCTGGTACTCT-3, r5, - GTGGTCGAGTTGGACTGGTTA-3,	60	164
*β-actin*	NM_031144.3	f5^,^- GGCATGTGCAAGGCCGGCTT − 3, r5^,^- TAGGAGTCCTTCTGACCCATA − 3^,^	58	116

### Histological examination and histomorphometrical analysis

Ovarian samples were taken from all groups of females at the conclusion of the trial, preserved for 24 h in a 10% neutral buffer formalin solution, and then prepared for histochemical and immunohistochemical staining. The samples were then cleaned in xylene, embedded, and blocked in liquid paraffin wax after being dehydrated in increasing concentrations of ethyl alcohol. A rotatory microtome was used to cut 5 μm slices of each sample, which were subsequently placed on coated glass slides for H&E staining ^49^  or positive glass slides for immunohistochemical staining of P53 and caspase-3.The immune histochemistry protocol was followed according to the protocol of Petrosyan, et al.^50^. Briefly, to suppress the action of endogenous peroxidase, the mounted positive glass slides were briefly dewaxed, rehydrated, and then treated with 0.3% hydrogen peroxide. Subsequently, in a humidified dark chamber, the sections were incubated with primary anti-P53 (Rabbit Recombinant Monoclonal, ab32049, 1:50 dilution, Abcam) and anti-caspase-3 (Rabbit Polyclonal, ab4051, 1:100 dilution, Abcam) overnight at 4 °C. Following a PBS rinse, the sections were incubated for one hour at room temperature with biotinylated secondary antibody (Goat Anti-Rabbit IgG, ab6721) and then for ten minutes with streptavidin. Mayer’s hematoxylin was used as a counterstain after DAB was used for five minutes to determine immune reactivity. Three rats were chosen at random from each group for histomorphometric analysis, and each rat had five non-overlapping sections (3 fields/section) viewed under a light microscope. The histomorphometrical analysis was applied using the ImageJ analysis tool (version 1.36, NIH, United States).

The following formula was used to determine the mean percentage of atretic follicles and deteriorated corpora lutea^8^.

$$\:\:\text{\%}\:\text{o}\text{f}\:\text{a}\text{t}\text{r}\text{e}\text{t}\text{i}\text{c}\:\text{f}\text{o}\text{l}\text{l}\text{i}\text{c}\text{l}\text{e}\text{s}\:=\frac{numer\:of\:atretic\:follicles}{Total\:number\:of\:follicles}x\:100$$

$$\:\text{\%}\:\text{o}\text{f}\:\text{d}\text{e}\text{g}\text{e}\text{n}\text{e}\text{r}\text{a}\text{t}\text{e}\text{d}\:\text{c}\text{o}\text{r}\text{p}\text{o}\text{r}\text{a}\:\text{l}\text{u}\text{t}\text{e}\text{a}=\:\:\frac{nnumber\:of\:degenerated\:corpora\:lutea}{total\:number\:of\:corpora\:lutea\:}x\:100$$

The type of each ovarian follicle in each section was categorized according to Flaws, et al.^51^. The mean number of each type of ovarian follicle was calculated to determine the folliculogenesis stages.

#### Statistical analysis

The Statistical Package for the Social Sciences (SPSS, version 17) was used to analyze the data with a one-way ANOVA with post-hoc Tukey’s test following normality verification via Shapiro-Wilk and Levene’s tests. GraphPad Prism version 8.0 (GraphPad Software, Inc., San Diego, California, USA) was used to create the figures. For every parameter, the experimental data were presented as means ± SEM. For each letter, the probability of (*p <* 0.05) was deemed significant.

## Results

### GC–MS analysis of Spirulina

**Fig. 2:**
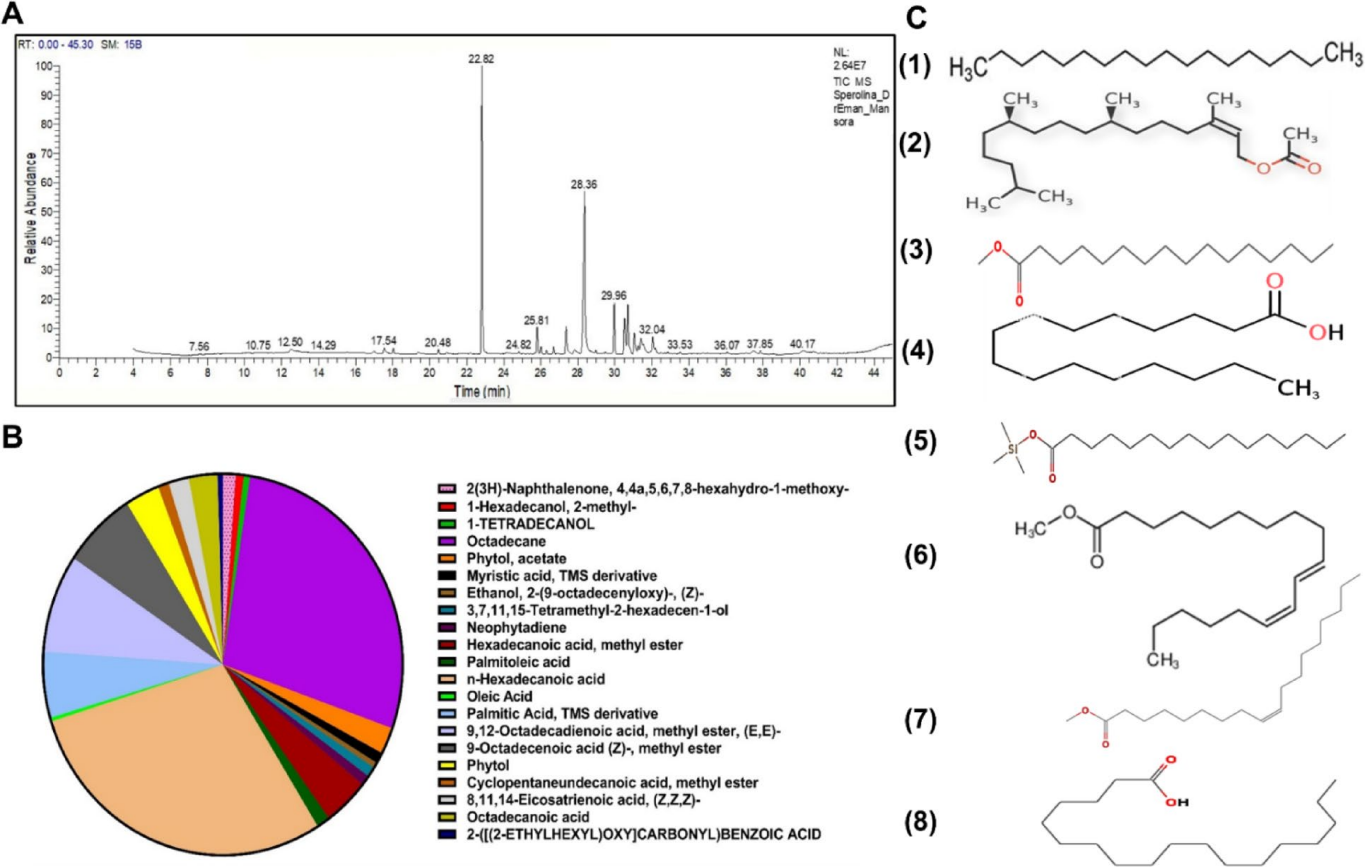
(A) Profile of GC-MS analysis for Spirulina. (B) Relative abundance of phenolic acids and flavonoids in Spirulina. (C) The chemical structure of the main identified components (1) Octadecane; (2) Phytol acetate; (3) Hexadecanoic acid methyl ester; (4) n-Hexadecanoic acid; (5) Palmitic Acid, TMS derivative; (6) 9,12-Octadecadienoic acid, methyl ester, (E, E); (7) 9-Octadecenoic acid (Z)-, methyl ester; (8) Octadecanoic acid.

The main content of Spirulina was n-Hexadecanoic acid (28.89%) and Octadecane (27.85%) (Fig. 2A, B). Additionally, the other main compounds were 9,12-Octadecadienoic acid, methyl ester, (E, E) (8.30%), 9-Octadecenoic acid (Z)-, methyl ester (6.58%), palmitic Acid, TMS derivative (5.56%), Hexadecanoic acid, methyl ester (4,08%), and phytol acetate (3.08%), **(**Fig. 2**)**.

### The interacting Polar residues, hydrophobic interactions, and binding affinities of either n-hexadecanoic acid or L-carnitine with SIRT1

**Fig. 3:**
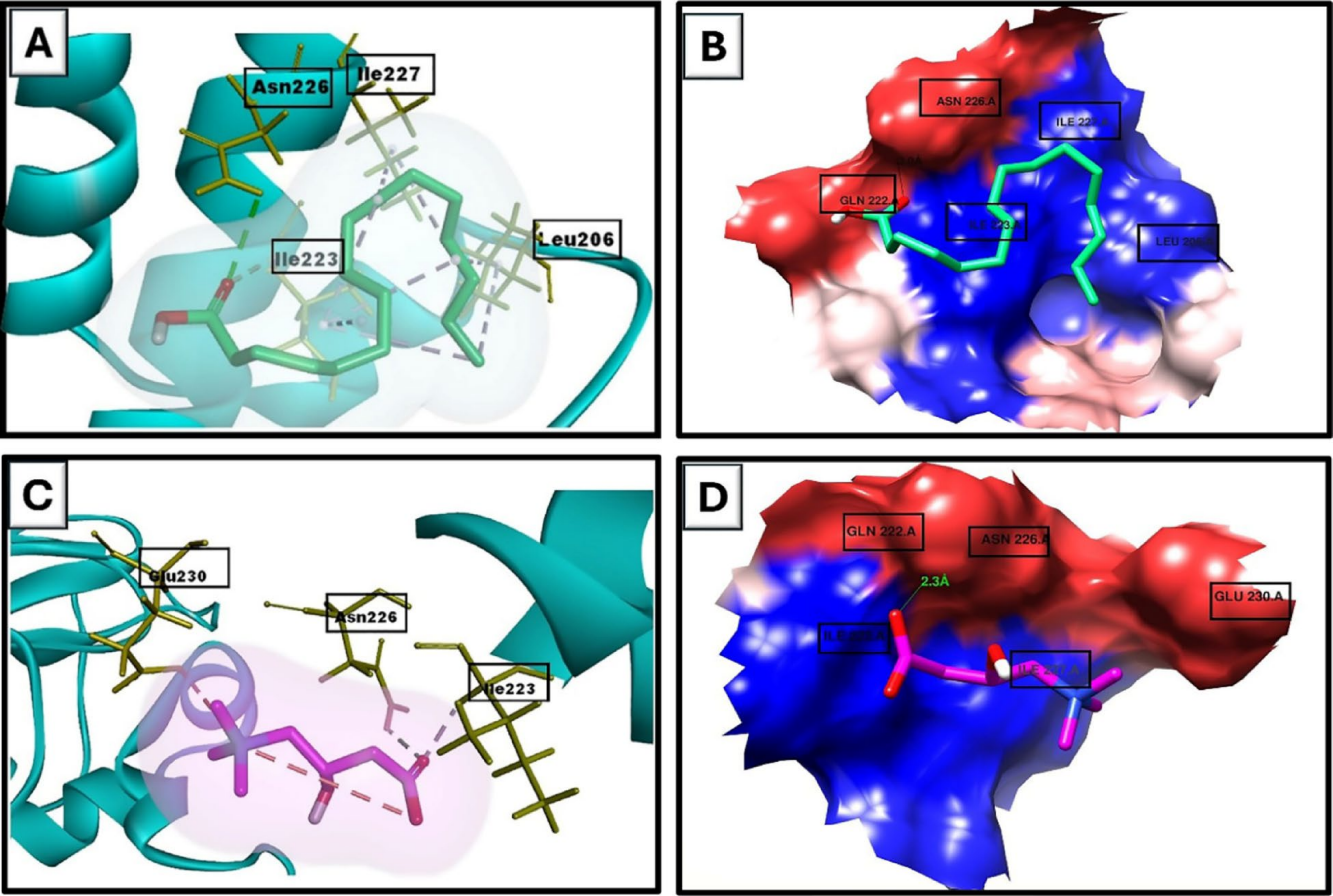
Molecular docking simulation revealing the binding between either n-hexadecanoic acid or L-carnitine with SIRT1. (A) Using Discovery Studio Visualizer, participate amino acids in the interaction between SIRT1 (PDB code: 4ZZJ) and n-hexadecanoic acid. (B) Using Chimaera, hydrophobic interactions among n-hexadecanoic acid and the SIRT1 pocket. (C) Utilizing Discovery Studio Visualizer, participate amino acids in the interaction between SIRT1 (PDB code: 4ZZJ) and L-carnitine. (D) Hydrophobic interactions between L-carnitine and the SIRT1 pocket using Chimera.

Our docking data revealed that n-hexadecanoic acid and L-car upregulate SIRT1. The binding affinities were − 3.8 kcal/mol and − 2.6 kcal/mol when n-hexadecanoic acid and L-car engaged with SIRT1 at the 4TQ location. Both n-hexadecanoic acid and L-car were able to form hydrogen bonds with ASN226. The output revealed that the binding of n-hexadecanoic acid with SIRT1 was encouraged by associations with LEU206, ILE223, and ILE227, which were hydrophobic. Also, L-car forms hydrophobic interactions with SIRT1 at the residues ILE223 and ILE227. The results of docking were illustrated in Fig. 3**and** Table 2.

**Table 2:** The interacting Polar residues, hydrophobic interactions, and binding affinities of either n-hexadecanoic acid or L-carnitine with SIRT1.

Molecular target and PDB code	Ligand	Hydrogen bond analysis		Amino acids involved in lipophilic analysis*	2D ligand/protein interaction	Binding affinity (kcal/ mol)
		Hydrogen bonds ligand/ Protein	Distance (Å)			
**SIRT1 (AZZJ)**	**n-hexadec-anoic acid**		3	LEU206, ILE223 and ILE227		-3.8
	**L-carnitine**		2.3	ILE223 and ILE227		**-2.6**

### Effect of SP and L-car on serum hormonal levels following DOX-mediated POI rats

**Fig. 4:**
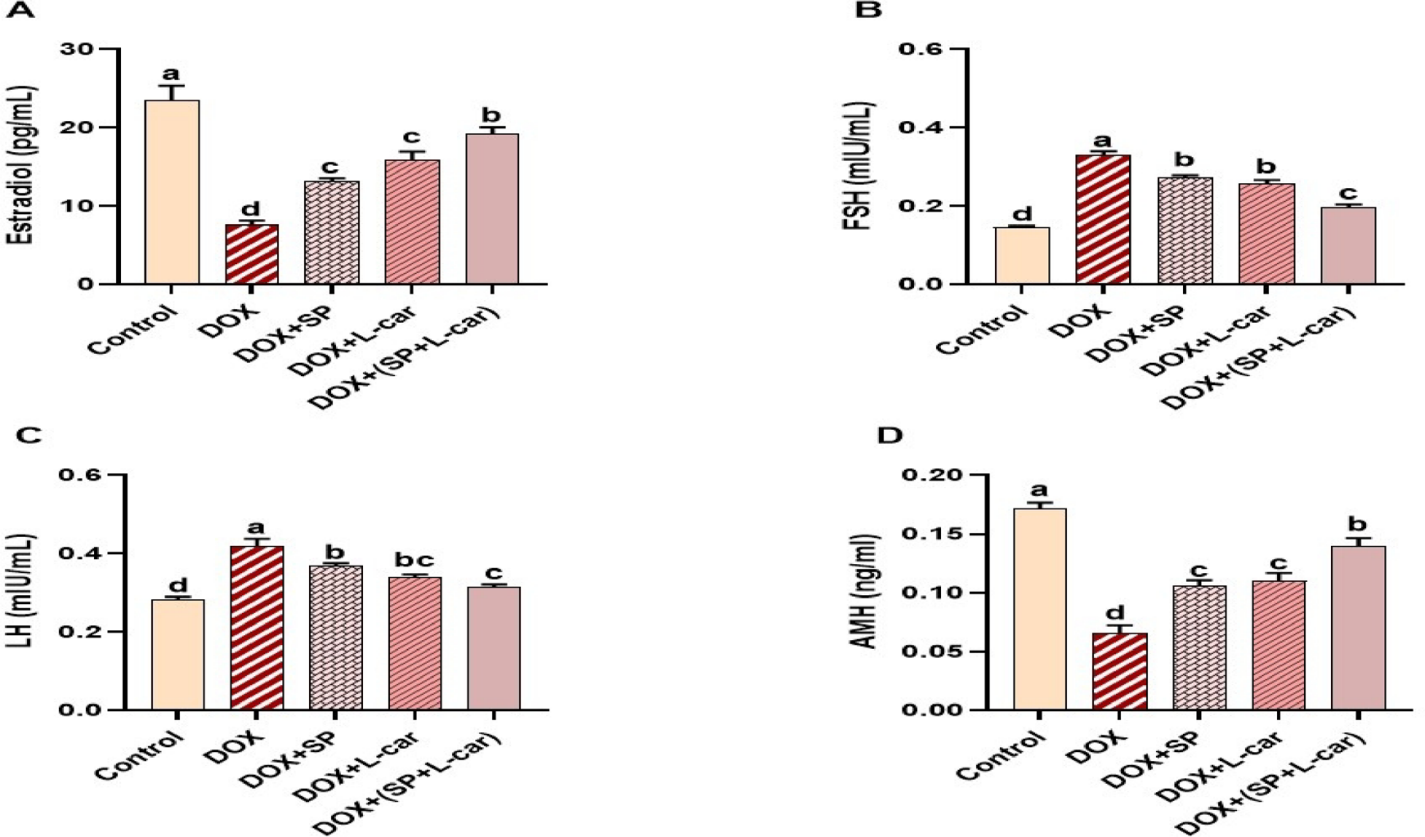
Palliative effects of spirulina (SP) and L-carnitine (L-car) on serum levels of estradiol (E2, A), follicle-stimulating hormone (FSH, B), luteinizing hormone (LH, C), and anti-mullerian hormone (AMH, D) following doxorubicin (DOX) mediated ovarian insufficiency in rats. Data are expressed as means ± SEM (*n* = 6). At *p* < 0.05, values denoted by distinct superscript letters are deemed significant.

To explore hormonal changes in DOX-intoxicated rats and the possible protective role of SP and L-car, the levels of E2, FSH, LH, and AMH were examined in the serum samples **(**Fig. 4A-D, **respectively)**. In comparison to the control group, rats exposed to DOX exhibited a significant (*p* < 0.05) reduction in E2 and AMH levels along with a significant (*p* < 0.05) elevation in the FSH and LH levels. Interestingly, the administration of SP, L-car, and their combination was able to reverse the changes in hormonal levels as presented in the DOX + SP, DOX + L-car, and DOX+(SP + L-car) groups, especially the DOX+(SP + L-car), unlike the DOX group, reflecting their protective role against ovarian toxicity induced by DOX.

### Effect of SP and L-car on ovarian oxidant/antioxidant molecules following DOX-mediated POI rats

**Fig. 5:**
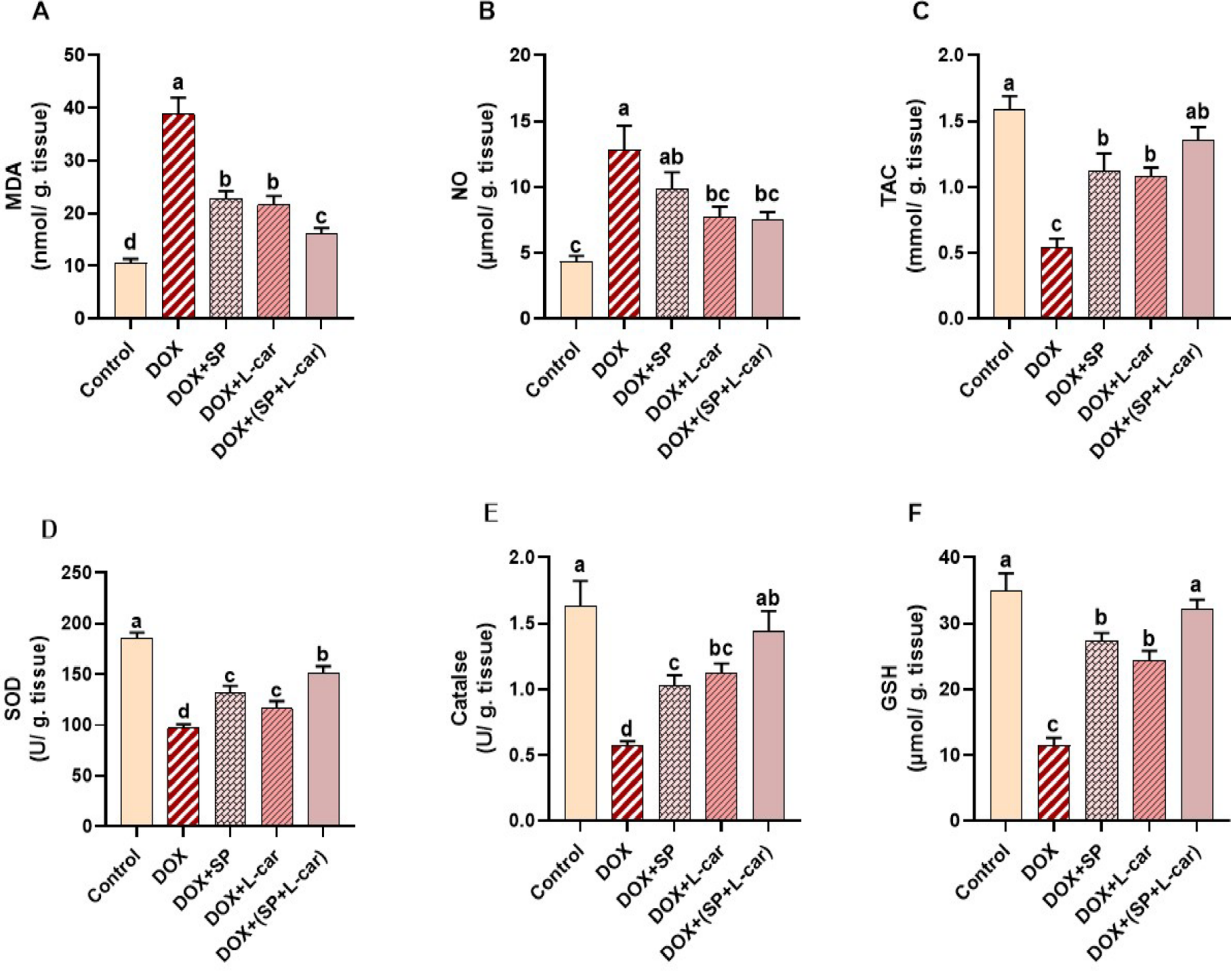
Palliative effects of spirulina (SP) and L-carnitine (L-car) on ovarian malondialdehyde (MDA; A), nitric oxide (NO; B), total antioxidant capacity (TAC; C), superoxide dismutase (SOD; D), catalase (CAT; E), and glutathione (GSH; F) following doxorubicin (DOX)-mediated ovarian insufficiency in rats. Data are expressed as means ± SEM (*n* = 6). At *p* < 0.05, values denoted by distinct superscript letters are deemed significant.

Figure 5 displays the ovarian redox state in response to SP and L-car treatments and ovarian insufficiency induced by DOX. In contrast to the group under control, greater (*p <* 0.05) ovarian MDA and NO levels were noticed in the model (DOX) group that considerably enhanced lipid peroxidation **(**Fig. 5A, B, **respectively)**. Aside from that, damaged ovarian tissues had a much lower amount of TAC, the enzymatic activities for SOD and CAT, and GSH level (*p <* 0.05) as opposed to the control **(**Fig. 5C-F, **respectively)**. Conversely, the administration of L-car alone or in combination with SP significantly (*p <* 0.05) inhibited the developed oxidative insults (MDA, NO) following the exposure to DOX. Similarly, SP alone significantly (*p <* 0.05) lowered the lipid peroxidation associated with DOX through decreasing MDA levels in ovarian tissue. On the other hand, relative to the untreated DOX group, the endogenous antioxidant molecules (TAC, SOD, CAT, GSH) in ovarian tissues were considerably (*p <* 0.05) increased by SP and L-car treatments, especially their combination.

### Effect of SP and L-car on mRNA expression of ovarian SIRT1 following DOX-mediated POI rats

**Fig. 6:**
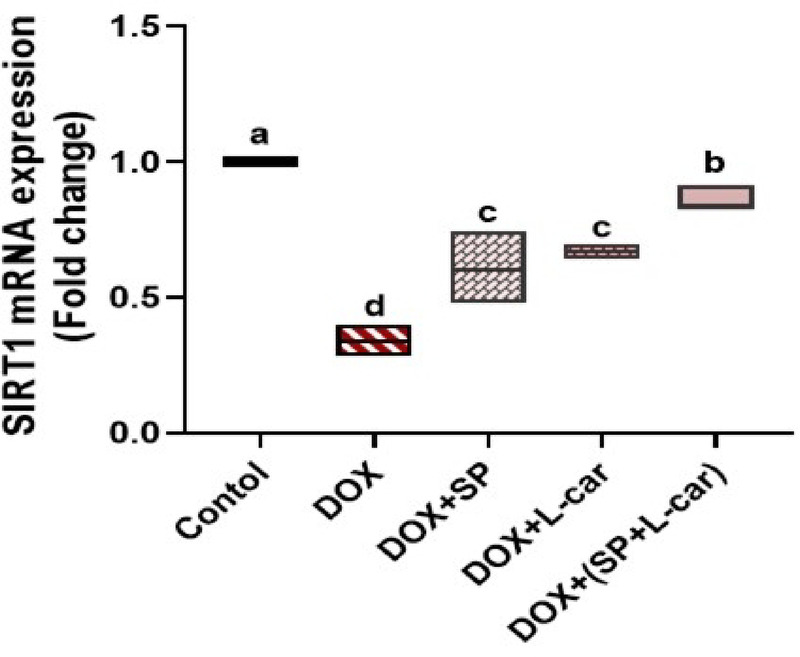
Palliative effects of spirulina (SP) and L-carnitine (L-car) on ovarian SIRT1 mRNA following doxorubicin (DOX)-mediated ovarian insufficiency in rats. Data are expressed as means ± SEM (*n* = 6). At *p* < 0.05, values denoted by distinct superscript letters are deemed significant.

SIRT1 levels were measured in ovarian samples as a master regulator of cytoprotective pathways in POI rats by the qRT-PCR method. DOX administration significantly decreased mRNA expression of ovarian SIRT1 compared to the control group (*p <* 0.05). Treatment with SP and/or L-car significantly increased ovarian SIRT1 levels. Importantly, administration of both SP and L-car in POI rats revealed greater (*p <* 0.05) effectiveness in enhancing SIRT1 mRNA expression (Fig. 6).

### Effect of SP and L-car on mRNA expression of ovarian steroidogenesis pathway following DOX-mediated POI rats

**Fig. 7:**
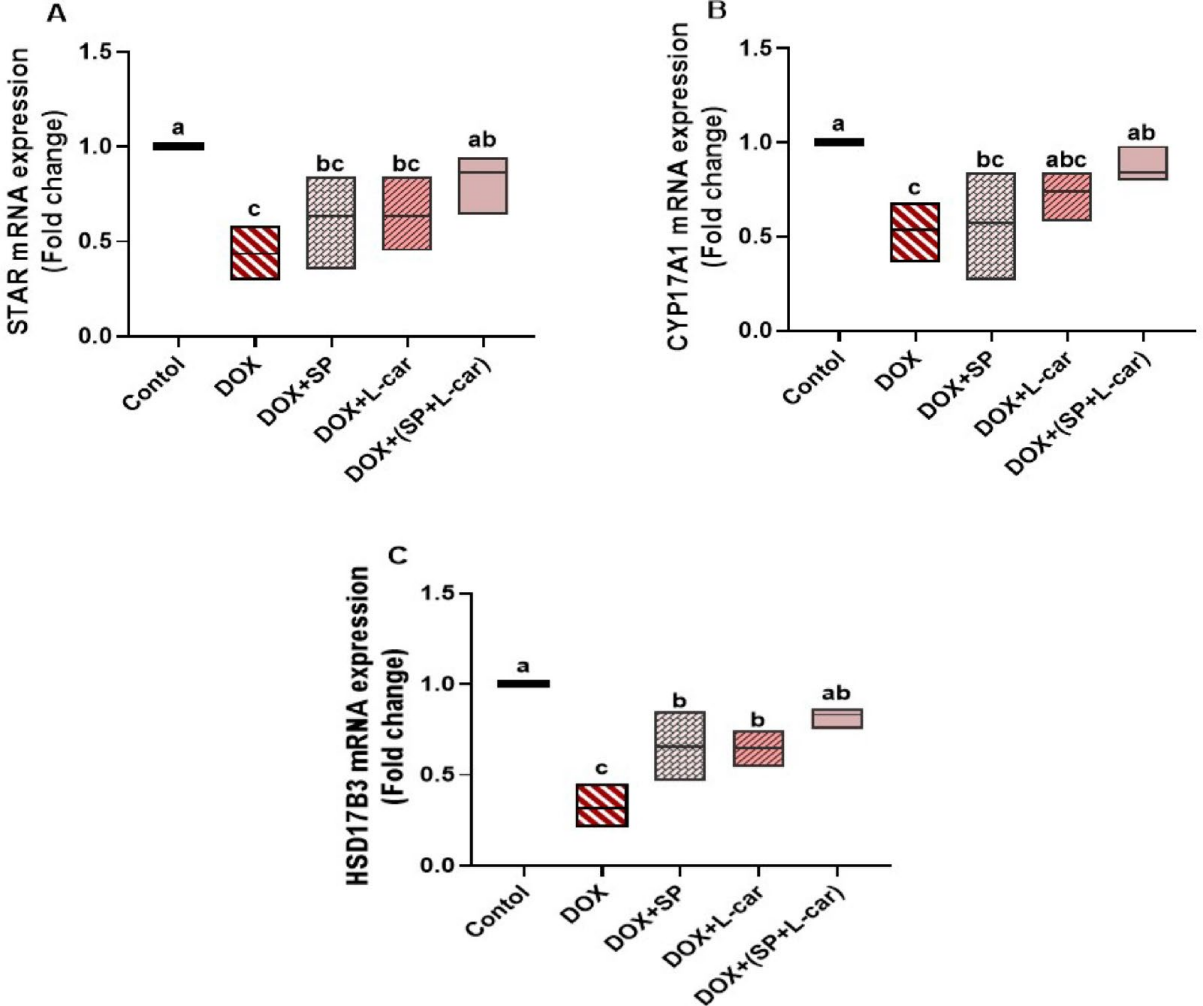
Palliative effects of spirulina (SP) and L-carnitine (L-car) on ovarian STAR mRNA (A), CYP17A1 mRNA (B), and HSD17B3 mRNA (C) following doxorubicin (DOX)-mediated ovarian insufficiency in rats. Data are expressed as means ± SEM (*n* = 6). At *p* < 0.05, values denoted by distinct superscript letters are deemed significant.

The current study’s findings showed that the DOX group’s relative mRNA expression of the ovarian STAR, CYP17A1, and HSD17B3 was significantly (*p* < 0.05) downregulated as compared to the control group. On the other hand, the relative mRNA expression of the genes described above was significantly (*p* < 0.05) higher in the treated groups than in the DOX group. Meanwhile, SP + L-car brought the expression of these genes back to nearly normal levels (Fig. 7A-C, **respectively**).

### Effect of SP and L-car on mRNA expression of ovarian Nrf2, mtDNA, and FOXO1 following DOX-mediated POI rats

**Fig. 8:**
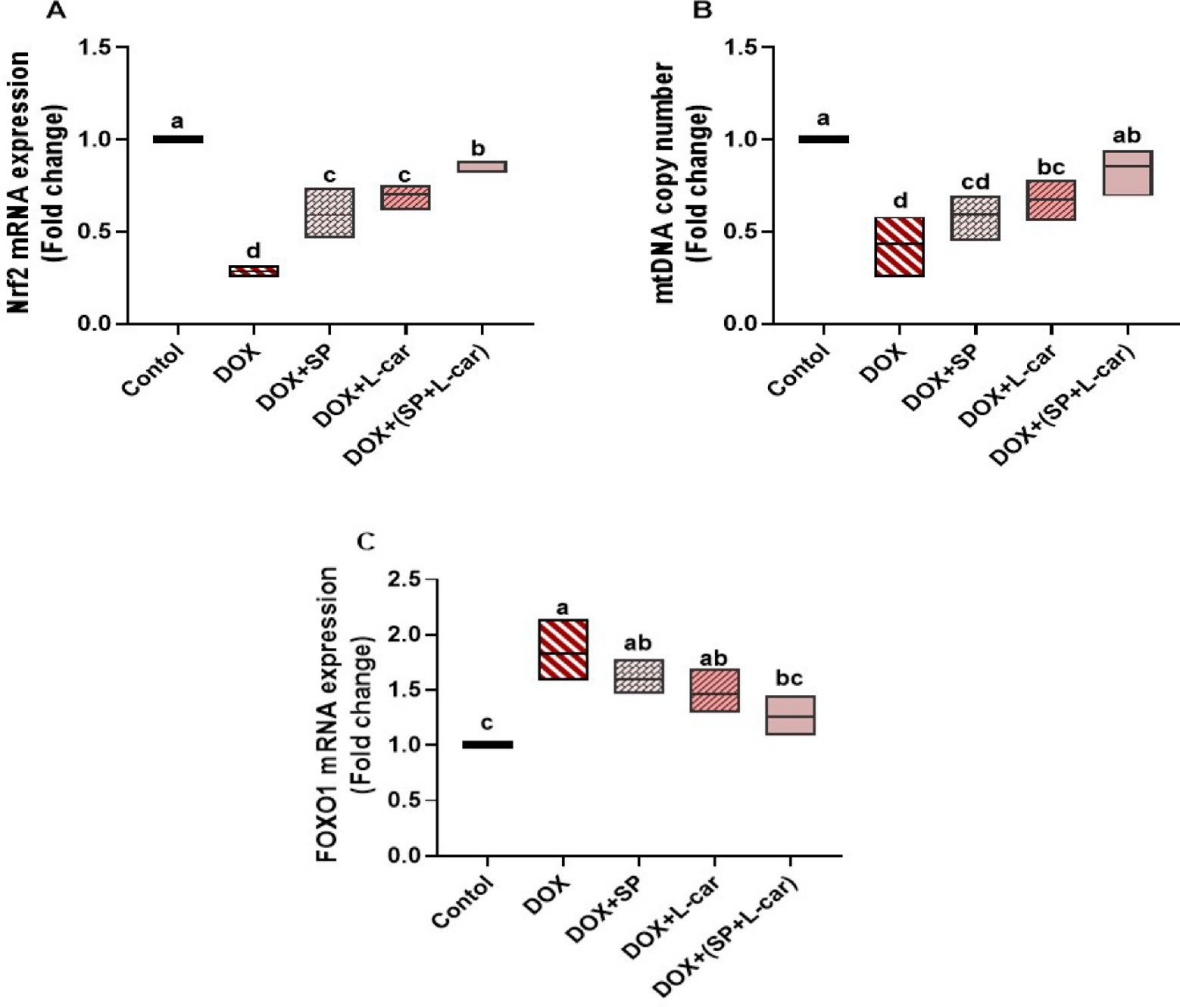
Palliative effects of spirulina (SP) and L-carnitine (L-car) on ovarian Nrf2 mRNA (A), mtDNA copy number (B), and FOXO1 mRNA (C) following doxorubicin (DOX)-mediated ovarian insufficiency in rats. Data are expressed as means ± SEM (*n* = 6). At *p* < 0.05, values denoted by distinct superscript letters are deemed significant.

In comparison to the control group, the DOX group’s ovaries had significantly (*p* < 0.05) lower levels of Nrf2 expression and mtDNA copy number, as well as a significant (*p* < 0.05) upregulation in the FOXO1 mRNA expression (Fig. 8A-C, **respectively)**. Conversely, administration of SP and/or L-car in POI rats significantly (*p <* 0.05) reversed all these outcomes in comparison to the DOX group. Also, delivering both SP and L-car to the DOX-treated rats revealed more attenuation against oxidative stress and apoptosis than either of the treatments alone.

### Effect of SP and L-car on mRNA expression of NF-κB/iNOS inflammatory signaling pathway following DOX-mediated POI rats

**Fig. 9:**
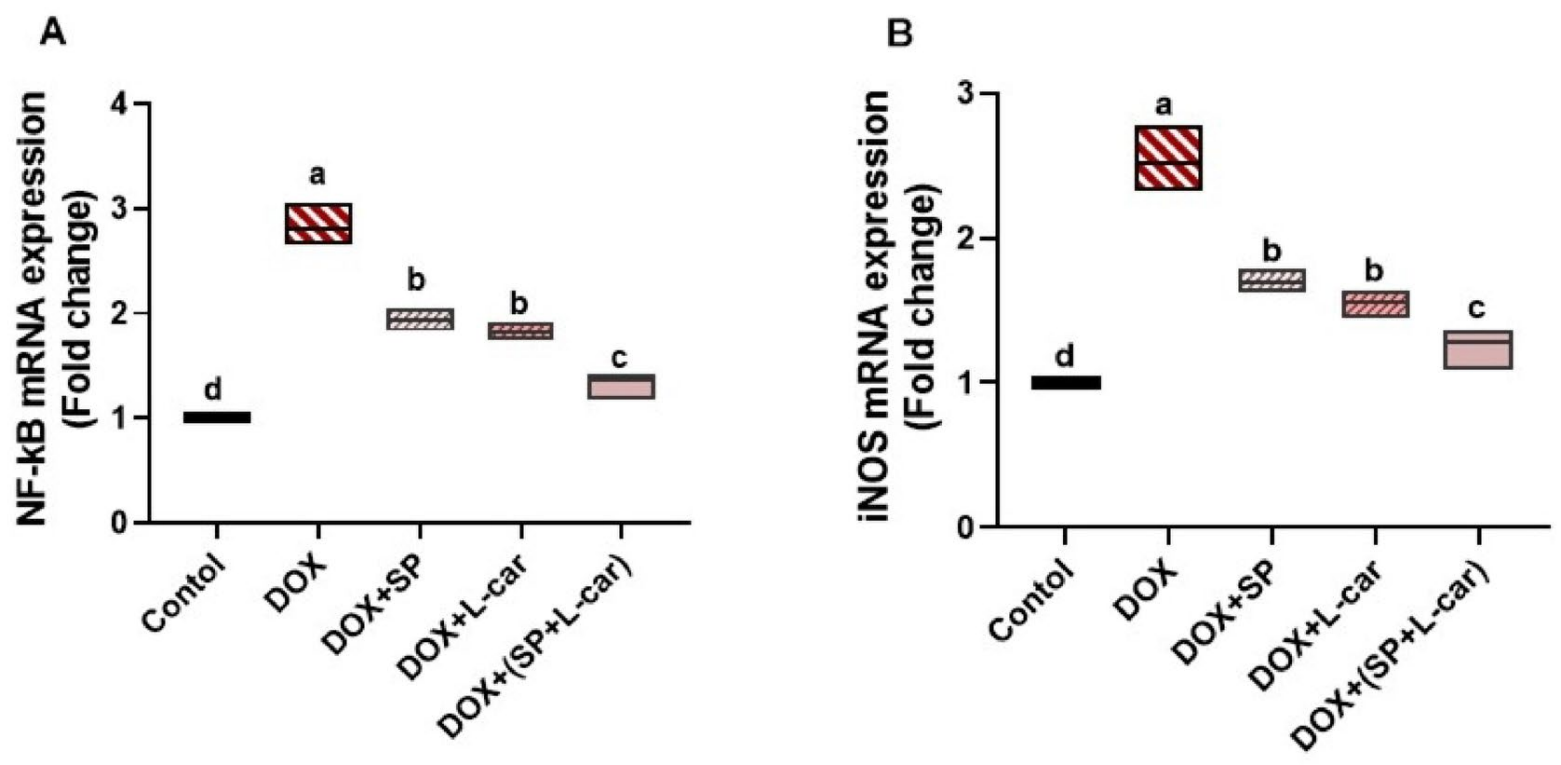
Palliative effects of spirulina (SP) and L-carnitine (L-car) on ovarian NF-κB mRNA (A) and iNOS mRNA (B) following doxorubicin (DOX)-mediated ovarian insufficiency in rats. Data are expressed as means ± SEM (*n* = 6). At *p* < 0.05, values denoted by distinct superscript letters are deemed significant.

The relative mRNA expressions of the NF-κB and iNOS were significantly (*p <* 0.05) upregulated in POI rats. However, treatment with SP and L-car significantly (*p <* 0.05) down-regulated the ovarian levels of these specific inflammatory biomarkers compared to rats from POI. On the other hand, SP + L-car induced a significant (*p <* 0.05) improvement in the relative expression of these biomarkers compared to either of the treatments alone (Fig. 9A, B, **respectively**).

### Effect of SP and L-car on ovarian histopathological findings and morphometry following DOX-mediated POI rats

**Fig. 10:**
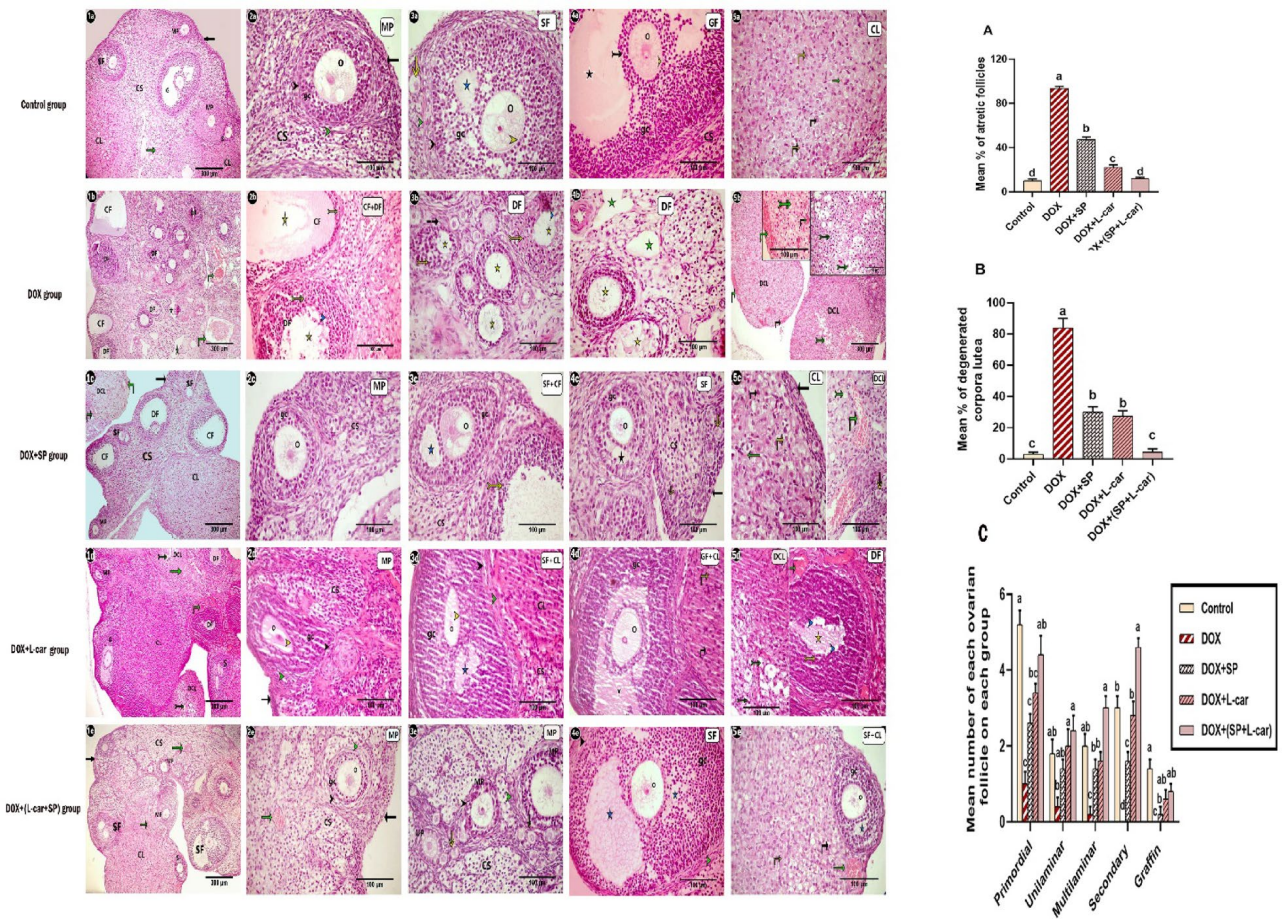
Photomicrograph of rat’s ovarian sections (cross section) stained with H&E showing different ovarian follicles; unilamellar primary follicle (yellow arrow), multilamellar primary follicles (MP), secondary follicles (SF) and mature graffin follicles (G) with oocytes (O), granulosa cells (GCs), Zona pellucida (yellow arrow head), theca interna (black arrow head) and theca externa (green arrow head), cortical stromal cells (CS), cumulus oophorous (black tailed arrow), normal blood vessels (green arrow), small fluid filled cavity (blue asterisk), large antrum (black asterisk), degenerated follicles (DF), degenerated granulosa cells (yellow tailed arrow), degenerated zona pellucida (blue arrow head), cystic follicles (CF), degenerated oocytes (yellow asterisk), dilated and congested blood vessels ( green vertical arrow), cortical area without cortical stromal cells (green asterisk), enlarged and congested blood vessels (green vertical arrow), normal corpus luteum (CL) with large (black vertical arrow) and tiny lutein cells (yellow vertical arrow), degenerated corpus luteum (DCL) with vacuolated and Pyknotic lutein cells that are dark (green tailed arrow).(A, B and C) representing histomorphometric analyses of the ovarian tissues.(A): representing mean % of atretic follicles.(B): representing mean % of degenerated corpora lutea.(C): representing mean number of each ovarian follicles in each group. Our findings are presented as mean ± SEM. The various letters (a, b, c, and d) reveal a significant difference among the experimental groups.

H&E-stained sections of the ovaries from the control group **(**Figs. 1a, 2, 3, 4, 5a and 10**)** exhibited outer cortex containing follicles at different stages of maturity and well-developed corpora lutea, and inner medulla composed of a well-vascularized loose connective tissue. Several stages of normal folliculogenesis were detected in the cortex in the form of primordial, unilaminar, and multilaminar primary, secondary, and Graafian mature follicles. Follicles contain one large oocyte with an euchromatic nucleus. They are surrounded by a typical zona pellucida and either one layer of flat granulosa cells in the primordial follicles, or one layer of cuboidal granulosa cells in the unilaminar primary follicles, or more than one layer of granulosa cells (GCs) in the multilaminar primary follicles and secondary follicles. Several antral cavities were detected between GCs in the secondary follicles. Graafian mature follicles contain a large antrum and eccentric oocytes surrounded by corona radiata. Follicles are surrounded by theca interna and externa and embedded within fibroblast-like cells and cortical stromal cells. The corpus luteum was identifiable with large and small lutein cells separated by small blood vessels.

On other hand, the sections of ovaries from the DOX group **(**Figs. 1b, 2, 3, 4, 5b and 10**)** showed notable degenerative and atrophic alterations both in ovarian follicles in all stages and corpora lutea, as the average percentage of atretic follicles **(**Fig. 10, A**)** and degenerated corpora lutea **(**Fig. 10, B**)** in this group was significantly (*p* < 0.05) increased in contrast to the control group. These atretic follicles appeared with the absence of the oocyte nucleus, disrupted granulosa cells, and irregular disruption in the zona pellucida. The lutein cells of degenerated corpora lutea appeared vacuolated with dark pyknotic nuclei, and the blood vessels between them were congested and dilated. In addition to that, the density of cortical stromal cells was markedly decreased, and vacuolated areas without stromal cells were detected between the atretic follicles. A typical follicular cyst of variable size in the cortex and dilated and congested blood vessels in the medullary region were also detected. The examination of ovarian sections from the DOX + SP **(**Figs. 1c, 2, 3, 4, 5c and 10**)**, DOX + L-car **(**Figs. 1d, 2, 3, 4, 5d and 10**)**, and DOX+(SP + L-car) **(**Figs. 1e, 2, 3, 4, 5e and 10**)** groups revealed histoarchitecture improvement when compared to the DOX group, as the mean % of atretic follicles **(**Fig. 10, A**)** and degenerated corpora lutea **(**Fig. 10, B**)** was significantly (*p* < 0.05) reduced compared to this group. The lowest % of atretic follicles and degenerated corpora lutea was detected in the DOX+(SP + L-car) group, followed by the DOX + L-car group and then the DOX + SP group, as the histoarchitecture of the DOX+(SP + L-car) group was more or less the same as the control group. The treatment with DOX + SP or DOX + L-car alone or concurrently with each other (DOX + SP + L-car) enhances the folliculogenesis as various kinds of ovarian follicles at different developmental stages appear bulging into the ovarian surface **(**Fig. 10, C**).**

### Effect of SP and L-Car on the protein expression of ovarian P53 and caspase-3 following DOX-mediated POI rats

**Fig. 11:**
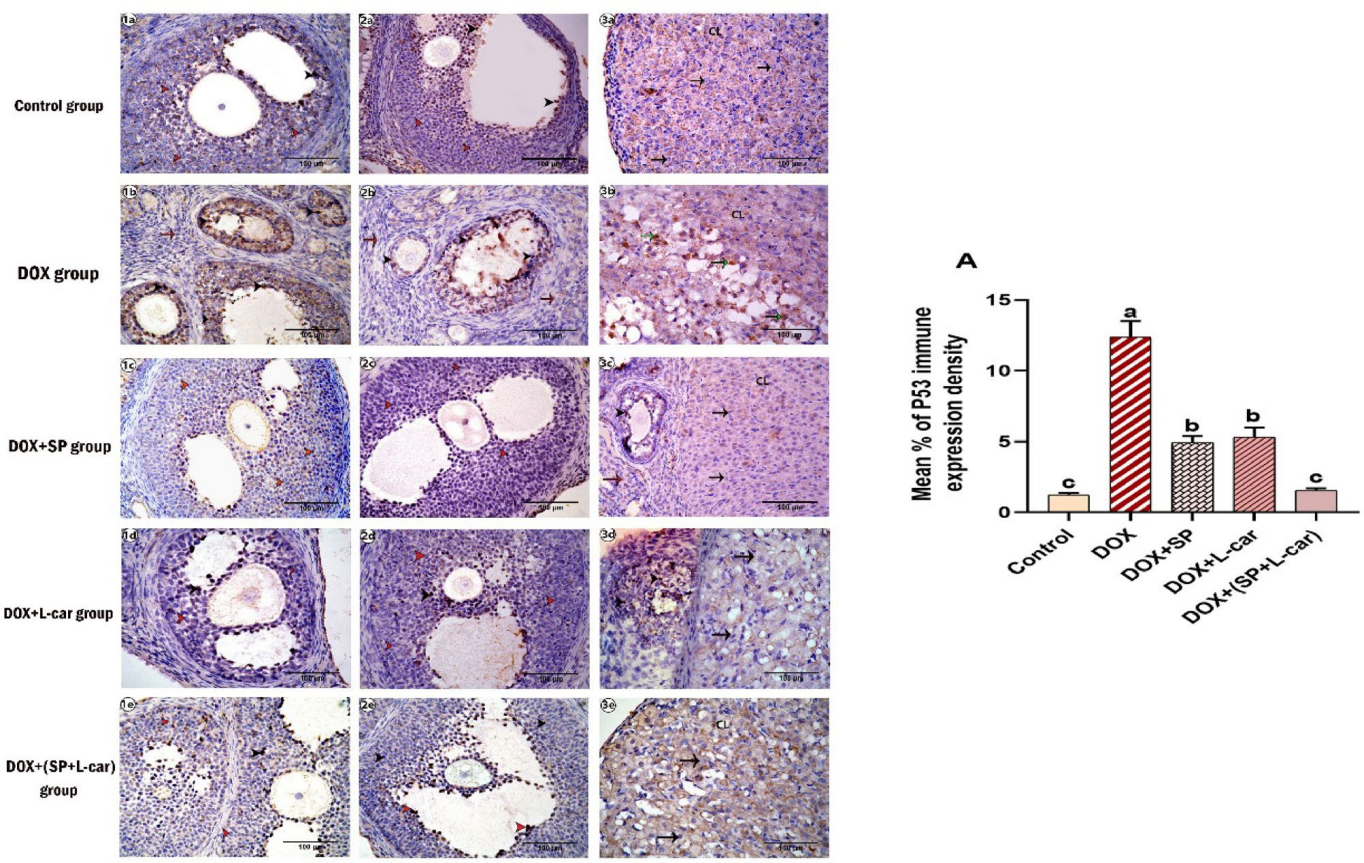
Photomicrograph of enzyme immunohistochemical staining of ovarian tissue for P53 showing granulosa cells (orange arrowhead), lutein cells (black arrow), and cortical stromal cells (orange arrow) with P53 negative immune expression. Granulosa cells (black arrowhead) and lutein cells (green arrow) with P53 positive immune expression. (A): representing the mean % of p53 immune expression density in different animal groups. Our data are expressed as mean ± SEM. The different letters (a, b and c) indicate marked differences between experimental groups.

Sections of ovaries from the control group **(**Figs. 1a, 2, 3a and 11**)** exhibited very weak P53-positive immune expression in the granulosa cells. Still, the density of P53 positive immune expression was significantly increased (*p* < 0.05) **(**Fig. 11, A**)** in the DOX group **(**Figs. 1b, 2, 3b and 11**)** compared to the control group. This reaction was moderately detected in the DOX + SP **(**Figs. 1c, 2, 3c and 11**)** and DOX + L-car **(**Figs. 1d, 2, 3d and 11**)** groups but significantly (*p* < 0.05) decreased compared to the DOX group. In the DOX+(SP + L-car) **(**Figs. 1e, 2, 3e and 11**)** group, the immune density of P53 was nearly the same as that in the control group.

**Fig. 12:**
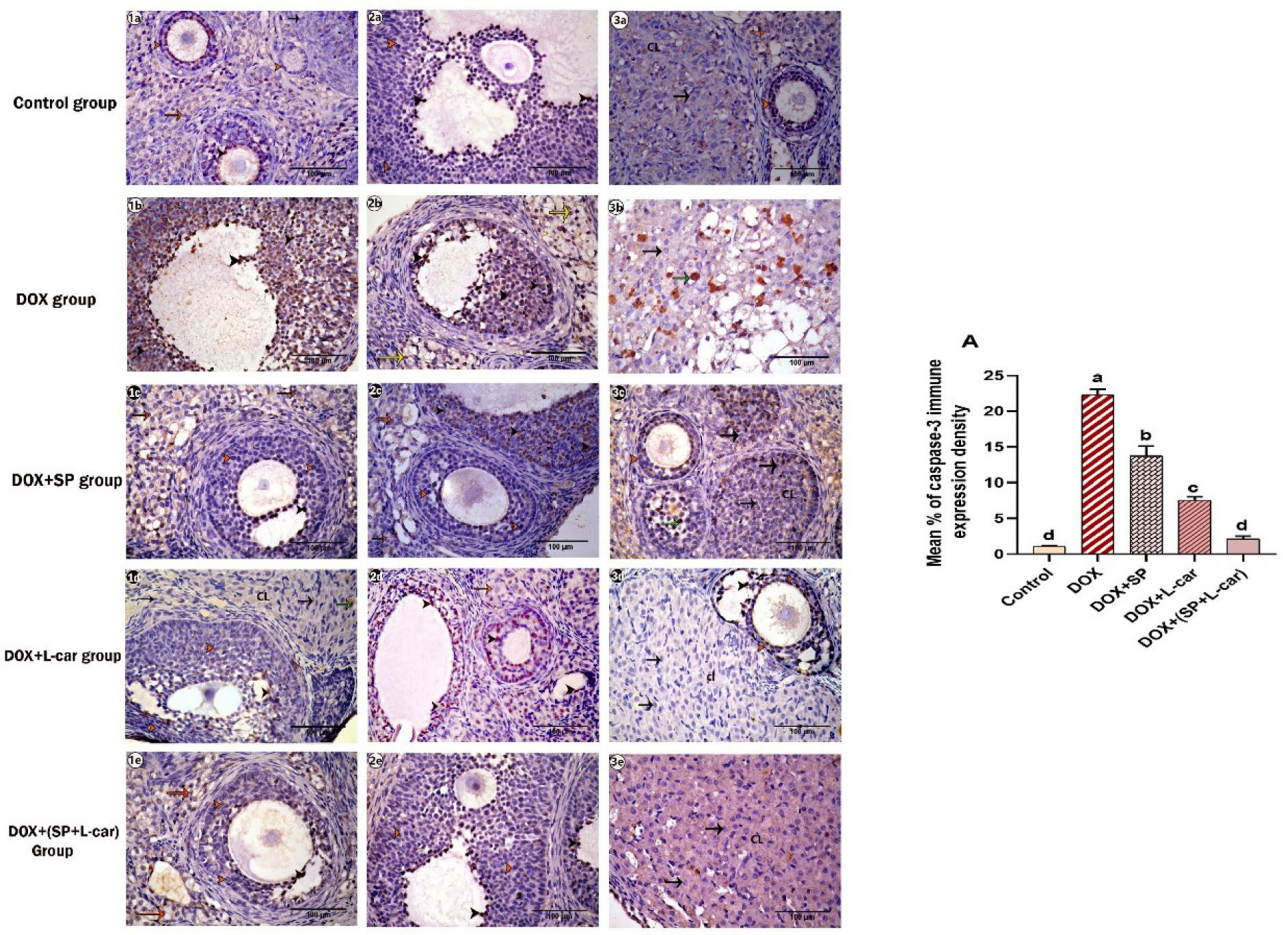
Photomicrograph of ovarian tissue stained with the enzyme caspase-3 immunohistochemically showing cortical stromal cells (orange arrow), granulosa cells (orange arrowhead), and lutein cells (black arrow) with caspase-3 negative immunological expression. Cortical stromal cells (yellow arrow), granulosa cells (black arrowhead), and lutein cells (green arrow) with caspase-3 positive immune expression. (A): representing the mean % of caspase-3 immune expression density in different animal groups. Our data are presented as mean ± SEM. The different letters (a, b, c, and d) show a noticeable change among experimental groups.

In the control group **(**Figs. 1a, 2, 3a and 12**)**, the immune expression of caspase-3 was hardly detected in the GCs of the ovarian follicles and corpora lutea; meanwhile, in the DOX group **(**Figs. 1b, 2, 3b and 12**)**, the immune density of caspase-3-positive cells was significantly (*p* < 0.05) **(**Fig. 12, A**)** increased compared to the control group. The immune density of caspase-3 positive cells was significantly decreased *(p < 0.05)* in the following three groups: DOX + SP **(**Figs. 1c, 2, 3c and 12**)**, DOX + L-car **(**Figs. 1d, 2, 3d and 12**)**, and DOX+(SP + L-car) **(**Figs. 1e, 2, 3e and 12**)** compared to the DOX group. The lowest expression of caspase-3 positive cells was detected in the DOX+(SP + L-car), as the mean expression of it was nearly the same in the control group.

## Discussion

Improvements in chemotherapeutics have gradually raised the survival rate of cancer patients in the recent decade, which has led to a greater focus on maintaining quality of life after cancer therapy^52,53^. Following cancer treatment, these women’s ability to preserve their ovarian reserve at reproductive age has a direct impact on any future infertility issues. Ovarian primordial follicular cells cannot regenerate, and aberrant death of these cells by chemotherapeutics leads to follicular dysfunction and oocyte atresia, which appears as POI^54^. Therefore, by shielding the ovaries, the additives are being researched to help young patient’s female cancer patients receiving emergency chemotherapy maintain their fertility.

A rat model of DOX-induced failure of ovaries was used in the experiment for this study: Serum levels of E2, FSH, LH, and AMH were measured in the current investigation to verify ovarian failure. This is consistent with research conducted by a few writers^7^ Who stated that blood tests used to determine the levels of the hormones, as mentioned earlier, were thought to be a trustworthy way to estimate fertility status and determine the ovaries’ reproductive lifespan.

The present investigation demonstrated that in rats given DOX, serum levels of E2 hormone secreted by the ovaries showed a significant decrease. In contrast, expression levels of FSH and LH released by the pituitary gland rise significantly to compensate for this loss, causing damage to normal ovarian tissue and germ cells and increasing the likelihood of developing POI in the ovary. These results concurred with Zhang et al.^55^ and Kim et al.^56^ who clarified that the chemotherapeutic drugs would harm the generation of ovarian GCs (OGCs). On the same ground, in contrast to the control group, the POI group’s steroidogenic genes, such as STAR, CYP17A1, and HSD17B3, were significantly downregulated, according to the study’s findings. This could be because oxidative stress causes an ovarian inflammatory and apoptotic microenvironment, which in turn inhibits and/or shuts off the ovarian steroidogenesis pathway^57,58^. Remarkably, the ovarian steroidogenesis-related genes listed above were restored in POI female rats by SP and/or L-car injection in line with. In ovarian failure, the ovaries generated little to no estrogen, which caused the pituitary and hypothalamus to lose their negative feedback mechanism. Consequently, the pituitary glands generated higher amounts of FSH and LH^59,60^.

Follicle development requires AMH, which is produced by the cells known as granulosa of developing follicles and utilized in the clinic as a screening and prognostic predictor of female fertility^61^. By preventing stimulation of primordial follicles and decreasing the antral follicles’ sensitivity to FSH throughout the recruitment phase of follicular dynamics, AMH adversely affects primordial follicle recruitment. For the ovary to have a certain number of primordial and developing follicles, AMH is essential^62^. According to our research, DOX therapy lowers AMH levels. It lessens its inhibitory influence on the recruitment of primordial follicles, which might cause the primordial follicular store to become overactive and POI. Fortunately, the low AMH expression brought on by DOX was up-regulated by SP and/or L-car therapy, supporting a rise in the function of the ovarian reserve of the ovary. Agreeing with the current work results, Almukainzi, et al.^63^ examined how SP counteracted cyclophosphamide-induced ovarian toxicity by stopping ovarian follicles from dying and re-establishing the gonadotropin hormone equilibrium, and Kalhori, et al.^64^ showed that lowering oxidative stress, inflammatory processes, and apoptosis in mice when the condition polycystic ovarian syndrome develops, L-car improves endocrine function and folliculogenesis.

Concomitantly, primordial follicle number counts more clearly show ovarian damage from treatment^65^. The current study’s light microscopic analysis revealed a significant atrophic and degenerative alteration in the corpora lutea and ovarian follicles in all stages, because DOX-intoxicated animals had a considerably larger mean proportion of atretic follicles and deteriorated corpora lutea than the untreated group. Nishi, et al.^8^and Wang, et al.^66^ demonstrated that severe damage to ovarian follicles at different stages of puberty was detected following DOX administration as well, and their numbers were significantly decreased. Another study has shown that the growth of follicles and the production of hormones in mice are adversely affected by even a low dose of DOX^67^. On the contrary, the continued treatment of DOX-intoxicated rats with SP or L-car implies that the ovaries have survived harm due to ovarian reserve preservation. Furthermore, their combination is multipotent, increasing the ovarian performance and reproductive potential of DOX-treated rats.

In this work, we investigated the mechanisms behind SP and L-car’s protective impact against DOX-induced ovarian damage. SP is one of the recognized potential reservoirs of antioxidants^68^. It is a good source of biologically active substances for the diet, such as essential fatty and amino acids, a high amount of protein (60–70%), minerals, carotenoids, vitamins, and pigments involved in photosynthetic processes^69,70^. The major component in the SP was n-hexadecanoic acid (palmitic acid, 28.89%), so the docking was performed by using n-hexadecanoic acid. L-car (3-aminobutyric acid) is a chemical that occurs naturally and is vital for bioenergetics activities^71^. It exhibits several biological functions, like antioxidant^72^anti-apoptotic and anti-inflammatory functions^73^. Our MD results demonstrated the ability of both n-hexadecanoic acid and L-car to form hydrogen bonds with the ASN226 residue of SIRT1, indication their ability to activate SIRT1 ^74^. This result came in agreement with Heo MyeongGang and Choung SeYoung^75^ and Radwan, et al.^35^who reported the ability of n-hexadecanoic acid and L-car to relieve high-fat diet-induced obesity and methotrexate induced nephrotoxicity through upregulation of SIRT1, respectively.

Recently, several studies have provided evidence for the use of SIRT1 as a potential treatment target and biomarker for POI. Loss of SIRT1 function in rats is due to overall follicle activation^76^. It was discovered that ovaries overexpressing SIRT1 had fewer atretic follicles along with more primordial follicles^77^. Along with this finding, in this research, we discovered that following DOX therapy, rats’ ovaries had lower levels of SIRT1 expression. Also, we discovered that lower SIRT1 levels hastened primordial follicle loss and follicular dormancy, which impacted the destiny of primordial follicles, and increased oxidative stress and harm to DNA, resulting in mitochondrial malfunction. Additionally, the current study’s findings demonstrated that SP, L-car, and their combination exhibited a significant expression of SIRT1, suggesting that SP and L-car may have a protective effect in the regeneration of ovarian tissues in our infertile rats.

The fundamental cause of DOX-induced ovarian failure is known to be increased formation of reactive oxygen species (ROS), and many free radical scavengers have been studied to prevent POI^78^. The results of the current investigation demonstrated that significant increases in MDA and decreases in TAC, SOD, CAT, and GSH were seen in DOX-induced ovarian oxidative stress. Additionally, the aforementioned endogenous antioxidant molecules were further depleted as a result of DOX-mediated inactivation of the Nrf2/antioxidant signaling pathway. The Nrf2, a redox-active signal protein that increases the antioxidant response and phase 2 detoxification responses in animals to control cellular redox equilibrium^79^. It is known that some of the downstream signal antioxidant enzymes of Nrf2 include SOD, CAT, heme oxygenase-1, glutathione reductase, and glutathione peroxidase^80^. These explain how DOX causes POI by speeding up the ovarian tissues’ lipid peroxidation, which makes the antioxidant system incapable of scavenging ROS. Consequently, by delaying oocyte maturation and triggering GCs’ death, high ROS buildup results in infertility^81^. Conversely, SP or L-car therapy increased the SOD and CAT antioxidant activity and GSH level while significantly decreasing the ovarian peroxidation marker (MDA). Additionally, POI animals who received both SP and L-car concurrently showed greater attenuation against oxidative stress than those that received either therapy alone. It was discovered that the radical scavenging action of the SP and L-car, in conjunction with the activation of endogenous antioxidant molecules through Nrf2 signaling, was responsible for their antioxidant efficacy^82,83^.

In line with the earlier finding, DOX administration in this investigation markedly elevated oxidative stress by blocking Nrf2 activation and lowering endogenous antioxidant enzyme levels^84^. It has been observed that oxidative stress induction is associated with reduced SIRT1 expression^85^. Our research made it clear that, in comparison to the control group, the DOX group’s ovaries downregulated SIRT1 expression, markedly elevated oxidative stress. Furthermore, it was noted that Nrf2 activation is amplified by SIRT1 signaling^86^. Therefore, the present finding of high expression of Nrf2 along with high SIRT1 expression in ovarian cells indirectly confirms the regulatory role of SIRT1 in protecting the GCs against ROS.

DOX has been linked to the production and accumulation of ROS in the mitochondria, which can damage mitochondrial DNA and impair the activity of the mitochondria^87^. Compared to normal rats, we explored changes in mtDNA copy number in the other studied groups. The existing POI rats showed lower mtDNA copy number. At the same time, a significant increase in mtDNA content was observed in ovarian cells after SP and L-car treatment in DOX-intoxicated rats. mtDNA is vulnerable to damage from oxidative stress, and a number of variables are thought to increase this vulnerability^88^. It has been proposed that a feedback process that compensates for mitochondrial flaws includes mutant mtDNA and a malfunctioning respiratory system, which raises ROS production in the mitochondria and induces more oxidative damage^89,90^. These alterations may be related to mitochondrial malfunction brought on by DOX administration. POI is thought to be significantly influenced by oxidative stress, which is connected to ovarian ageing and mitochondrial failure^91^. However, by increasing SIRT1 accumulation in the ovary, SP and L-car could attenuate DOX-induced mtDNA damage in ovarian cells, subsequent mitochondrial dysfunction, and resulting apoptosis in preantral follicles, which was consistent with the earlier observation^92^.

Along with the findings of the oxidative stress and its associated mitochondrial malfunction and other earlier investigations, we have reason to believe that the DOX caused a significant reduction in E2. Following DOX therapy in rats, one of the most noteworthy events was the considerable decrease in E2, which indicated that the OGCs were less able to produce E2. It’s probable that DOX eventually reduces OGCs’ capacity to produce and release E2 by destabilizing mitochondria, the site of steroidogenesis^58,93^. Steroid hormones control the development of the follicle that is dominant as well as the secondary follicle’s atresia process^94^so it is crucial to sustain steroid hormone synthesis in DOX-treated rats in order to prevent premature aging. Several studies have suggested that SIRT1 regulates mitochondrial integrity in estrogen-producing cells through amelioration of mitochondrial stress^21^. Consequently, SIRT1 contributes to the overall growth of the ovarian follicles as it is necessary for the control of steroid production. Additionally, SIRT1 controls the activity of GCs to regulate the release of ovarian steroid hormones^95^. In view of the above, the notion that SIRT1 might preserve the integrity of mitochondrial function under oxidative stress brought on by chemotherapy is supported by the capacity of SP or L-Car to improve mitochondrial stress-induced disturbed steroidogenesis markedly.

In line with the earlier research, the current investigation discovered that DOX-induced ovarian toxicity was connected to increased levels of ovarian NF-κB, iNOS, and NO activity, in contrast to the untreated group^13^. However, in contrast to the DOX-only group, SP or L-car therapy dramatically reduced the previously indicated parameters. These numbers were returned to nearly normal status by their combination. NO was found to be a part of the pathogenesis of POI^96^. By turning on iNOS, DOX can have cytotoxic effects by causing NO production in ovarian tissue^97^. The peroxynitrite radical, which damages cells, is created when too much NO combines with a superoxide anion to oxidise and nitrate macromolecules, including GSH, proteins, lipids, and DNA. Furthermore, too much NO depletes intracellular GSH, making the body more vulnerable to oxidative stress^98^.

Among the most significant transcription factors that have been shown to be vulnerable to oxidative stress is NF-κB^99^. iNOS is one of the imperative target genes of NF-κB that is responsible for adjusting NO levels within the tissues^100–102^. Therefore, the inhibition of NF-κB/iNOS may be beneficial in reducing ovarian damage^103^. The crucial role of NF-κB and NO in the adverse effects seen with DOX treatment is well documented in several studies^104,105^. SIRT1 targets NF-κB directly, and by deacetylating it, it can modulate the amount of acetylation of NF-κB, which in turn controls the transcription level of iNOS and other inflammatory factors^106^. Therefore, by inhibiting NF-κB, iNOS, and NO activities through SIRT1 activation, SP or L-car’s protective impact would be linked to a decrease in the formation of oxidative free radicals and pro-inflammatory mediators. It was previously documented that SP exhibited anti-inflammatory and antioxidant properties against methotrexate-induced neurotoxicity^107^. Additionally, it has been demonstrated that preventative L-car therapy lowers the inflammatory responses to TNF-α and NF-κB in gastrointestinal toxicity caused by cisplatin^108^.

ROS can promote cell death through apoptotic signaling activation^109^. Through excessive ROS production, DOX has been shown to promote apoptotic signaling. This can simultaneously suppress anti-apoptotic signal proteins and activate proapoptotic factors^110^. DOX has an effect on primordial follicles after prolonged treatment, either by causing DNA damage or by excessively recruiting these follicles^66^. Martirosyan, et al.^111^ and Morgan, et al.^112^ in their studies on inhibitors of apoptosis in a trial to protect the ovary after DOX therapy concluded that DOX diminished primordial follicular reserve through the induction of apoptosis of GCs and oocyte which is parallel to the findings of the present histological sections of rat ovaries immunohistochemical stained with antiP53 and anticaspase-3 bodies. We discovered that the injection of DOX increased the percentage of their immunological density and improved the immune expression of P53 and caspase-3.

The P53 protein is essential for controlling how cells react to DNA damage and can prevent cytochrome c from being released, which can then trigger ovarian apoptosis^113^. DOX has been shown to enhance P53 protein expression, hence initiating apoptosis^114^. Stimulation of caspases, which play a key role in regulating apoptosis-associated chemical and cellular changes, is the most immediate trigger of apoptotic death^115^. It has been proposed that caspase-3 may play a role in the ovaries’ physiological follicular atresia^116^. It has been proposed that caspase-3 may play a role in the ovaries’ physiological follicular atresia^117^. The follicles may be more susceptible to apoptosis brought on by external cues, such as chemotherapy drugs, because of this natural cellular machinery. This characteristic could be linked to the increased incidence of POI after chemotherapy^118^. On the other hand, co-treatment with SP and L-car successfully decreased the regions of P53 and caspase-3 immunoexpression in rats given DOX, indicating that SP and L-car had anti-apoptotic properties in vivo. It has recently been shown that SP decreased the expression of P53 and caspase-3 in mice with chronic alcohol-induced liver damage^119^. Also, the antiapoptotic effects of L-car demonstrated by Soliman, et al.^120^ and Elsayed, et al.^121^. Interestingly, according to our results, SP and L-car pretreatment, because of their capacity to scavenge ROS and thereby lower apoptotic ovarian cell death, seem to offer protection against the various DOX-induced cell death pathways.

The present study’s findings supported current theories, as the POI group displayed a marked upregulation of ovarian FOXO1 expression along with a substantial reduction of SIRT1 expression. This was followed by an upregulation of pro-apoptotic markers P53 and caspase-3, which clearly reflects SIRT1’s negative regulatory role. Consequently, earlier research showed a connection between elevated FOXO1 expression and apoptosis^122^. In mammalian ovaries, the FOXO1 protein regulates atresia and follicular growth by promoting the induction of apoptosis in OGCs^123^. The suppression of ovarian FOXO1 expression effectively reversed DOX-induced follicular demise, resulting in a rise in the reserve of primordial follicles; this could provide a novel treatment option for DOX-associated POI. According to a study, apoptosis is believed to be suppressed by SIRT1-induced deacetylation of FOXO1. SIRT1 deacetylates FOXO1 to protect mitochondria from oxidative stress, which limits FOXO1’s potential to cause cell death^124^. Intriguingly, SP and L-car significantly reduced FOXO1 expression in the POI rat model, together with P53 and caspase-3 downregulation. This demonstrated that SP and L-car, through increasing SIRT1 expression, inhibited the pro-apoptotic function of FOXO1 ^125^.

**Fig. 13:**
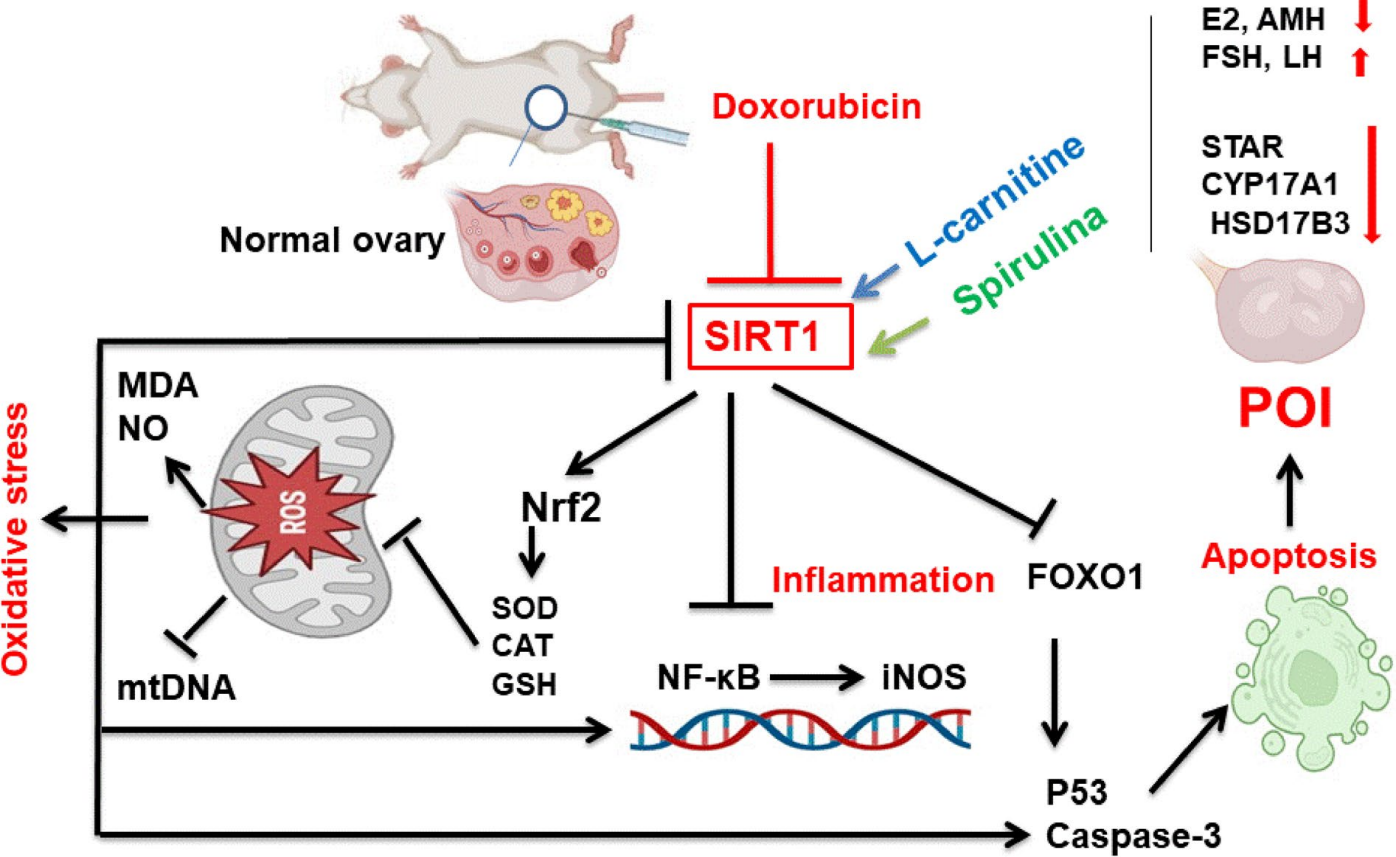
Graphical abstract shows different SIRT1 signaling pathways mediated by spirulina and L-carnitine against POI.

Taken together, according to earlier research, SIRT1 can shorten the lifetime of cells through deacetylating the P53 protein, along with decreasing its transcriptional activity^126^. Furthermore, NF-κB also has a role in controlling apoptosis^127^and using NF-κB, SIRT1 regulates the expression of anti-apoptotic genes^17^. As seen above, SIRT1 can influence apoptosis by regulating the degree of NF-κB deacetylation, which consequently impacts the toxic damage caused by certain toxicants. However, the inflammatory response is the primary mechanism by which these participate in the harmful effects process of toxicants through the SIRT1/NF-κB pathway^128^. The different SIRT1 signaling pathways mediated by spirulina and L-carnitine against POI were illustrated in Fig. 13.

Although this study provides valuable insights into the protective effects of spirulina and L-carnitine on ovarian function in a rat model of premature ovarian insufficiency, several limitations should be acknowledged. The use of a rat model, although widely accepted for preliminary biomedical research, may not entirely replicate the complex pathophysiological processes of premature ovarian insufficiency in humans. Consequently, the translational relevance of the observed effects of spirulina and L-carnitine requires further validation in human clinical trials. Furthermore, the study was limited to short-term assessments, focusing primarily on biochemical, histological, and selected molecular markers. Functional reproductive outcomes such as estrous cyclicity, mating behavior, fertility rates, and long-term preservation of ovarian reserve were not evaluated. The investigation also employed single doses and a fixed duration of treatment, which may not capture the full therapeutic potential or optimal dosing regimens of spirulina and L-carnitine. In addition, while molecular docking provided mechanistic insights, in vitro validation of the proposed SIRT1-mediated pathways was not performed. The absence of comprehensive safety and toxicological evaluations further limits the scope of clinical applicability. These limitations highlight the need for future studies incorporating long-term, dose-dependent investigations, in vitro confirmation of signaling pathways, reproductive outcome assessments, and safety profiling to fully elucidate the potential of spirulina and L-carnitine as therapeutic agents in the management of chemotherapy-induced premature ovarian insufficiency.

## Conclusion

In this study, the repair of impaired ovarian function and fertility through the regeneration of granulosa cells and oocytes, as well as the restoration of hormone profiles that support the use of antioxidants. Both SP and L-car attenuated DOX-induced ovarian oxidative damage, subsequent ovarian mtDNA expression, and activation of downstream inflammatory and apoptotic pathways, particularly lowering P53 and caspase-3 protein expressions in preantral follicles, thus preventing ovarian insult and follicle demise and extending the post-chemotherapy fertile period. As promising prophylactic ovoprotective agents, SP and L-car can be used as chemotherapy adjuvants, reducing the adverse health effects of early menopause after cancer therapy.

## Data Availability

The datasets used and/or analysed during the current study are available from the corresponding author on reasonable request.
